# Cerebrospinal Fluid Glucose and Lactate: Age-Specific Reference Values and Implications for Clinical Practice

**DOI:** 10.1371/journal.pone.0042745

**Published:** 2012-08-06

**Authors:** Wilhelmina G. Leen, Michèl A. Willemsen, Ron A. Wevers, Marcel M. Verbeek

**Affiliations:** 1 Department of Neurology, Donders Institute for Brain, Cognition and Behaviour, Radboud University Nijmegen Medical Centre, The Netherlands; 2 Department of Pediatric Neurology, Donders Institute for Brain, Cognition and Behaviour, Radboud University Nijmegen Medical Centre, The Netherlands; 3 Department of Laboratory Medicine, Radboud University Nijmegen Medical Centre, The Netherlands; California Pacific Medicial Center Research Institute, United States of America

## Abstract

Cerebrospinal fluid (CSF) analysis is an important tool in the diagnostic work-up of many neurological disorders, but reference ranges for CSF glucose, CSF/plasma glucose ratio and CSF lactate based on studies with large numbers of CSF samples are not available. Our aim was to define age-specific reference values. In 1993 The Nijmegen Observational CSF Study was started. Results of all CSF samples that were analyzed between 1993 and 2008 at our laboratory were systematically collected and stored in our computerized database. After exclusion of CSF samples with an unknown or elevated erythrocyte count, an elevated leucocyte count, elevated concentrations of bilirubin, free hemoglobin, or total protein 9,036 CSF samples were further studied for CSF glucose (n = 8,871), CSF/plasma glucose ratio (n = 4,516) and CSF lactate values (n = 7,614). CSF glucose, CSF/plasma glucose ratio and CSF lactate were age-, but not sex dependent. Age-specific reference ranges were defined as 5–95^th^ percentile ranges. CSF glucose 5^th^ percentile values ranged from 1.8 to 2.9 mmol/L and 95^th^ percentile values from 3.8 to 5.6 mmol/L. CSF/plasma glucose ratio 5^th^ percentile values ranged from 0.41 to 0.53 and 95^th^ percentile values from 0.82 to 1.19. CSF lactate 5^th^ percentile values ranged from 0.88 to 1.41 mmol/L and 95^th^ percentile values from 2.00 to 2.71 mmol/L. Reference ranges for all three parameters were widest in neonates and narrowest in toddlers, with lower and upper limits increasing with age. These reference values allow a reliable interpretation of CSF results in everyday clinical practice. Furthermore, hypoglycemia was associated with an increased CSF/plasma glucose ratio, whereas hyperglycemia did not affect the CSF/plasma glucose ratio.

## Introduction

Cerebrospinal fluid (CSF) analysis is an important tool in the diagnostic work-up of neurological disorders like infection or inflammation of the central nervous system, intracranial hemorrhage and inherited metabolic disorders. It is therefore remarkable that little consensus exists amongst different laboratories about the reference ranges for CSF glucose and lactate [1]. CSF glucose is decreased in, amongst others, patients with bacterial meningitis and leptomeningeal carcinomatosis, usually in combination with other abnormal CSF parameters, such as an elevated leucocyte count, total protein or lactate concentration [2]–[4]. Low CSF glucose, often in combination with low CSF lactate, can also be found in patients with GLUT1 deficiency syndrome, a treatable genetic metabolic disorder that is caused by impaired glucose transport into brain [5]. CSF lactate can be elevated in several disorders, such as subarachnoidal hemorrhage, bacterial meningitis [3], cerebral hypoxia [6], status epilepticus [7], and inborn errors of metabolism [8]. Age-specific reference values are needed for an accurate interpretation of the results of CSF analysis and differential diagnosis. The main objective of this study was to define age-dependent reference values for CSF glucose, CSF/plasma glucose ratio and CSF lactate for everyday clinical practice. Furthermore, we aimed to determine the correlations between CSF glucose or CSF/plasma glucose ratio and plasma glucose in order to gain a better understanding of the dynamics of glucose transport of glucose into brain under physiological as well as pathological (i.e. hypo- and hyperglycemic) conditions.

## Methods

### Literature study

We performed an extensive literature search using PubMed for studies on the subject of reference values for CSF glucose, CSF/plasma glucose ratio and CSF lactate by using MeSH terms (cerebrospinal fluid, analysis, glucose, CSF/plasma glucose ratio, lactate, reference value). Furthermore, we studied the relevant references mentioned in the articles.

### CSF samples

**Figure 1:**
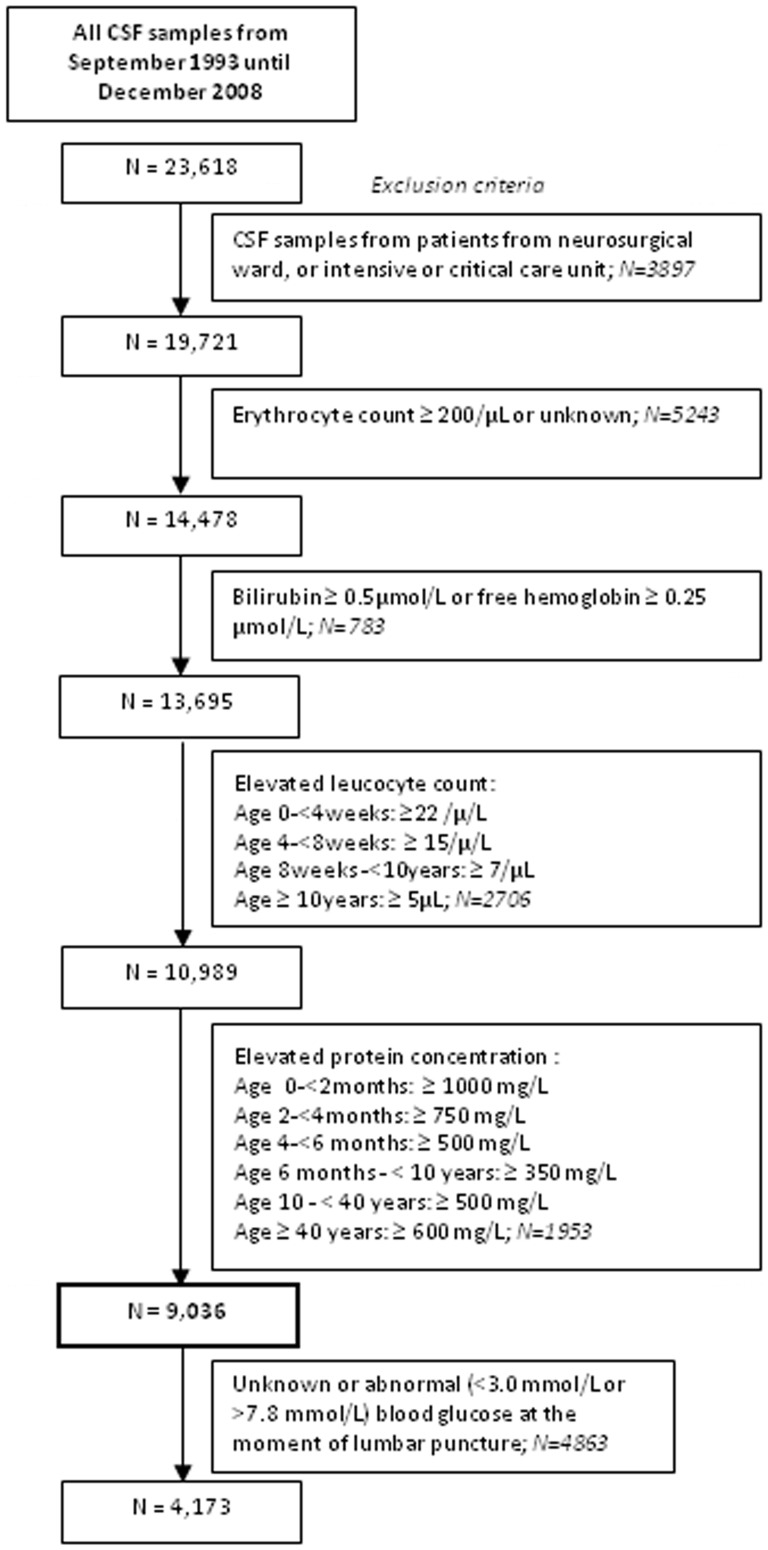
Flow chart of inclusion of CSF samples. N – number of CSF samples.

In September 1993 The Nijmegen Observational CSF was started. Results of all CSF samples that were analyzed at the laboratory of our tertiary referral hospital, the Radboud University Nijmegen Medical Centre in Nijmegen, The Netherlands (www.neurochemistry.nl) were systematically collected and stored in our computerized database. This study was approved by the local Medical Ethical Committee, METC Arnhem – Nijmegen. Since laboratory data had routinely been stored in the hospital database and were anonymously processed for the purpose of this study, the METC waived the need for patient informed consent. CSF samples that were analyzed between September 1993 and December 2008 (n = 23,618) were included. To define reference values for glucose and lactate in CSF obtained by lumbar puncture we have excluded (1) all CSF samples from patients from the neurosurgical ward or intensive care units (where CSF is often obtained from ventricular shunts); and CSF samples with (2) an erythrocyte count ≥200/µL or unknown erythrocyte count; (3) a bilirubin concentration ≥0.5 µmol/L and/or a free hemoglobin concentration ≥0.25 µmol/L; (4) an elevated leucocyte count; or (5) an elevated total protein concentration (Fig. 1). We have used strict exclusion criteria for leucocyte count and total protein concentration based on age-specific reference values as given in figure 1 [9]–[12]. In a previous study we identified all patients with GLUT1DS in our CSF database (n = 3) [13]. These patients were excluded from the present study. We finally included 9,036 CSF samples for further analyses. Patient population consisted of 49.7% males and 50.3% females. The population was predominantly Caucasian.

CSF glucose was determined in 8,871 CSF samples, CSF/plasma glucose ratio in 4,518 samples and CSF lactate concentrations in 7, 614 CSF samples. To determine whether multiple lumbar punctures from the same patient influenced our results, we have in second instance also studied the CSF glucose (n = 7,245) and CSF lactate (n = 6,856) concentrations if only the first CSF sample of each patient was included.

**Figure 2:**
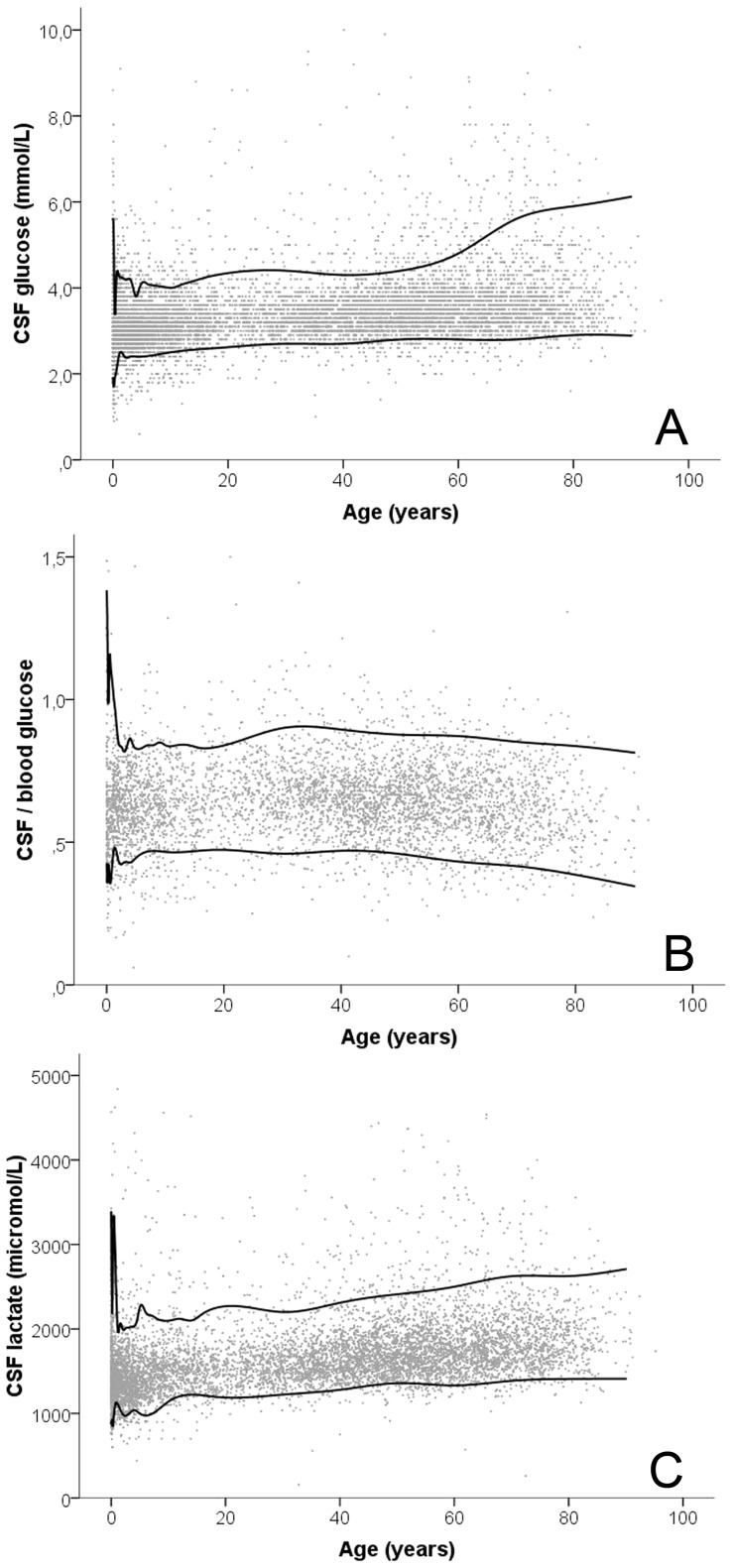
Age-specific CSF glucose, CSF/plasma glucose ratio and CSF lactate values. (A) CSF glucose concentration in 8,871 samples. CSF samples with CSF glucose >10.0 mmol/L (n = 4) are not shown; (B) CSF/plasma glucose in 4,516 samples. CSF samples with CSF/plasma glucose >1.5 (n = 5) are not shown; (C) CSF lactate concentration in 7,614 samples. CSF samples with CSF lactate >5000 µmol/L (n = 22) are not shown. Lines indicate 5^th^ and 95^th^ percentile values.

In second instance, the 5–95^th^ percentile ranges were determined after exclusion of (1) CSF samples from patients with hypoglycaemia (defined as plasma glucose <3.0 mmol/L), hyperglycemia (defined as plasma glucose >7.8 mmol/L) or unknown plasma glucose level at the moment of lumbar puncture (Fig. 1); (2) CSF samples with a clearly elevated CSF lactate (arbitrarily defined as >3000 µmol/L).

### CSF analysis

Lumbar punctures were performed in everyday clinical practice, thus on any time of the day, using standard procedures, and preferably after a ‘fasting’ period of at least 4–6 hours. CSF was collected in polypropylene tubes and was immediately transported after withdrawal to the adjacent laboratory. The CSF samples were analyzed within two hours after sample collection. After cell count, CSF was centrifuged at 860 g at room-temperature and glucose, lactate, total protein and blood pigments concentrations were analyzed the same day.

CSF glucose was measured by the hexokinase method [14]. CSF glucose was measured linearly until 10.9 mM with a limit of detection (LOD) of 0.056 mM, a cients of variation (CV) of <1.6% and a recovery of 97–100%.

The CSF lactate concentration was determined by the enzymatic conversion of NAD into NADH, measured at 340 nm, in the presence of lactate dehydrogenase. CSF lactate was measured linear until 13.32 mM with a LOD of 0.020 mM, cients of variation (CV) <1.2% and a recovery of 98–100%.

Total protein concentration in CSF was determined with the Lowry reaction and absorbance is measured at 720 nm. Lactate, glucose and total protein were analyzed with an automated analyzer (Mira Plus; ABX, Eindhoven, The Netherlands). The laboratory participates in external quality control programs of Instand (Düsseldorf, Germany) for the quality control of the glucose, lactate and total protein analyses. Results are available upon request. CSF erythrocytes and leucocytes count were determined manually in a Fuchs-Rosenthal counting chamber. Assay methods were completely validated if the method was altered during the inclusion period (e.g. change in detection device) and were evaluated according to the QC-system (ISO 15189).

### Plasma glucose analysis

The allowed time frame for parallel sampling of plasma and CSF was 30 minutes. Glucose was measured in plasma by the hexokinase method [14]. Venous blood was sampled in NaF-EDTA tubes and transported on room temperature.

### Statistical analysis

Statistical analyses were performed using SPSS version 20.0 (SPSS INC, Chicago, IL, USA). Patients were divided into age groups which are commonly used in daily clinical practice (as used by MeSH, Pubmed) (Table 1). We included all 9,036 CSF samples of which 8,871 samples were studied for CSF glucose, 4,516 samples for CSF/plasma glucose ratio and 7,614 samples for CSF lactate values. Reference values for CSF glucose, CSF/plasma glucose ratio and CSF lactate were based on the 5–95^th^ data-observed percentile ranges. 95% Confidence Intervals were based on Bootstrap Percentiles (based on 1000 bootstrap samples).

**Table 1:** Age-specific reference values for CSF glucose, CSF/plasma glucose ratio and CSF lactate.

Age		CSF glucose	CSF/plasma glucose	CSF lactate
		*mmol/L*		*mmol/L*
**Neonate**	0–4 weeks	1.9–5.6	0.42–1.10 (1.38)*	0.9–2.5 (3.4)^‡^
**Infant**	4–8 weeks	1.7–5.6 (5.1)*	0.36–1.20	0.9–2.2
	2–6 months	1.9–4.9 (3.9)*	0.39–1.10	1.0–2.2 (3.3)^‡^
	6–12 months	2.4–4.9 (4.3)*	0.44–1.05	1.1–2.2
**Toddler**	1–3 years	2.4–4.2	0.44–0.84	1.0–2.0
**Preschool child**	3–4 years	2.4–3.8	0.43–0.86	1.0–2.0
**School child**	4–10 years	2.5–4.0	0.45–0.84	1.1–2.1
**(Pre)adolescent**	10–18 years	2.6–4.3	0.47–0.83	1.2–2.2
**Young adult**	18–30 years	2.7–4.4	0.46–0.90	1.2–2.2
**Adult**	30–50 years	2.8–4.4	0.46–0.88	1.3–2.4
**Middle aged**	50–60 years	2.8–4.2 (4.8)*	(0.43) 0.48–0.87*	1.3–2.5
**Aged**	60–80 years	2.9–4.4 (5.6)*	(0.42) 0.46–0.84*	1.4–2.6
	≥80 years	2.9–4.5 (6.1)*	(0.35) 0.42–0.81*	1.4–2.7

Reference values are based on the 5^th^ to 95^th^ percentile values. The subgroups from the original data (see Tables S1,S2,S3) were clustered into age groups which are commonly used in daily clinical practice (as used by MeSH, Pubmed).

* Reference range after exclusion of CSF samples of patients with an unknown or abnormal plasma glucose (<3.0 mmol/L or >7.8 mmol/L) at the moment of lumbar puncture (only represented if >10% different from to the original value). Numbers between brackets represent values without correction for plasma glucose. ‡Reference range after exclusion of CSF samples with CSF lactate >3000 µmol/L (only represented if >10% different from the original value). Number between brackets represents upper limit without exclusion of CSF samples with CSF lactate >3000 µmol/L.

## Results

### Literature study

Reference ranges for CSF glucose, CSF/plasma glucose ratio and CSF lactate based on studies including large numbers of CSF samples were not available. Most studies had been performed in newborn or young infants and were based on a small number of patients (i.e. <350 patients) and only a few had been performed in patients without an explicit neurological disorder. We have summarized the results of our literature study in Table 2. In all studies CSF glucose was measured by the hexokinase method and CSF lactate was determined enzymatically.

**Table 2:** Summary of literature search for reference ranges of CSF glucose, CSF/plasma glucose ratio and CSF lactate.

Reference	N	Inclusion criteria	Age	CSF glucose concentration*(mmol/L)*		CSF/plasma glucose	CSF lactate concentration*(mmol/L)*	
				*As reported*	*Converted to P5-95*		*As reported*	*Converted to P5-95*
**Knight et al., 1981**	314	Healthy children	1 wk–16 yrs	Mean 3.63; P2.5-97.5: 2.56–5.54	2.72–4.54	-	Mean: 1.75; P2.5-97.5: 0.98–3.17	1.11–2.39
**Vamosi et al., 1983**	144	Cisternal CSF specimens of fasted adults free from organic brain disease	16–69 yrs	-	-	-	Mean ± 2SD: 0.68–2.1	-
**Ahmed et al., 1992**	108	Noninfected term neonates	0–30 dys	Mean ± SD: 2.8±0.7	1.65–3.95	Mean: 0.62	-	-
**Bonadio et al., 1992**	75	Term febrile infants with negative cultures	0–8 wks	Mean ± SD: 2.56±0.56	1.64–3.48	-	-	-
**Cameron et al., 1993**	100	Children with normal CSF glucose, protein and cell count	Children (not further specified)	Range: 3.3–5.5	-	-	Range: 0.5–3.2	-
**Benoist et al., 2003**	197	Hospitalized children	0–15 yrs	-	-	-	Median:1.5; SD 0.255 Range:0.6–2.2	1.08–1.95

N - number of CSF samples included in study; wk(s) – week(s); dys – days; yrs- years; P – percentile.

### CSF glucose concentrations

CSF glucose concentrations were measured in 8,871 out of 9,036 (98%) CSF samples. CSF glucose ranged from 1.0 to 11.9 mmol/L (mean 3.42 mmol/L; SD 0.769). CSF glucose concentrations were age-dependent with remarkably low 5^th^ percentile values until the age of 6 months (1.7–2.0 mmol/L) (Fig. 2A and Table S1). After the age of 6 months the CSF glucose 5^th^ percentile values gradually increased over the entire age range studied (from 2.4 to 2.9 mmol/L). CSF glucose 95^th^ percentile values were highest in neonates (5.6 mmol/L) and lowest in patients aged 3–6 months (3.9 mmol/L) and 3–4 years (3.8 mmol/l). Above the age of 4 years a gradual increase in the CSF glucose 95^th^ percentile was seen (from 4.0 to 6.1 mmol/L). After exclusion of CSF samples from patients with an abnormal or unknown plasma glucose level at the moment of lumbar puncture, the CSF glucose 5^th^ percentile value did not show a change of >10% of the original value. CSF glucose 95^th^ percentile values, however, did change more than 10% in patients aged 4–8 weeks, 3–12 months and ≥60 years after the application of these exclusion criteria. Numbers were unaffected in the other age groups.

### CSF/plasma glucose ratio

Plasma glucose was measured in 4,516 out of 9,036 (50%) paired samples and ranged from 0.80 to 40.4 mmol/L (mean 5.5 mmol/L; SD 1.98). An elevated plasma glucose (>7.8 mmol/L) was found in 338 samples (7.5%). Plasma glucose was elevated in 13.5% of the samples from patients older than 50 years of age, compared to 3.7% in samples from patients younger than 50 years of age. A lowered plasma glucose (defined as <3.0 mmol/L) was found in 24 samples (0.5%) of patients aged 0–78 years (mean 19.4 years; SD 22.9).

The CSF/plasma glucose ratio is described in Fig. 2B and Table S2. CSF/plasma glucose ratio ranged from 0.17 to 2.03 (mean 0.65; SD 0.14). Low CSF/plasma glucose ratio 5^th^ percentile values were seen below the age of 6 months (0.36–0.42) and in children aged 3–4 years (0.43) compared to neighboring age groups. Above the age of 4 years the CSF/plasma glucose 5^th^ percentile values gradually increased from 0.44 to 0.46 until the age of 40 years, with a continuous decrease afterwards to 0.35. CSF/plasma glucose ratio 95^th^ percentiles were high in patients below the age of 12 months (1.03–1.38).

After exclusion of CSF samples from patients with abnormal (defined as >7.8 mmol/L or <3.0 mmol/L) or unknown plasma glucose level, CSF/plasma glucose ratio 95^th^ percentiles were >10% lower compared to the original value in patients below the age of 4 weeks, whereas >10% higher 5^th^ percentile values were found in patients older than 50 years of age. Numbers were unaffected in the other age groups.

### CSF lactate concentrations

CSF lactate concentrations were measured in 7,614 out of 9,036 (84%) CSF samples. CSF lactate ranged from 0.16 to 12.5 mmol/L (mean 1.7; SD 0.48) CSF lactate showed a clear age-dependent pattern (Fig. 2C and Table S3). CSF lactate 5^th^ percentile concentrations were, like CSF glucose, remarkably low in (young) children (0.86–1.14 mmol/L) and increased with age. CSF lactate 95^th^ percentile values were particularly high in neonates (3.4 mmol/L) and low in children aged 6 months to 10 years (2.0–2.1 mmol/L). After the age of 18 years a gradual increase of the 95^th^ percentile value was seen from 2.2 to 2.7 mmol/L. Exclusion of CSF samples with a CSF lactate value of >3000 µmol/L did not lead to a change of >10% of the original value of the 5^th^ of 95^th^ percentile value, except in patients under the age of 4 weeks and in patients aged 3–6 months.

### Age-specific reference values

If multiple CSF samples from the same patient were excluded and only the first CSF sample of each patient was included, the 5^th^ and 95^th^ percentile ranges for CSF glucose (n = 7,245), CSF/plasma glucose ratio (n = 3,862) and CSF lactate (n = 6,856) values did not change >10% compared to the original value). Age-specific reference values were therefore based on the 5^th^ and 95^th^ percentile ranges of all CSF samples (8,871 CSF samples for CSF glucose; 4,516 samples for CSF/plasma glucose ratio and 7,614 samples for CSF lactate) (Table 1). Reference ranges for CSF glucose, CSF/plasma glucose ratio and CSF lactate did not differ between males and females.

### Relation between plasma glucose, CSF glucose and CSF/plasma glucose ratio

**Figure 3:**
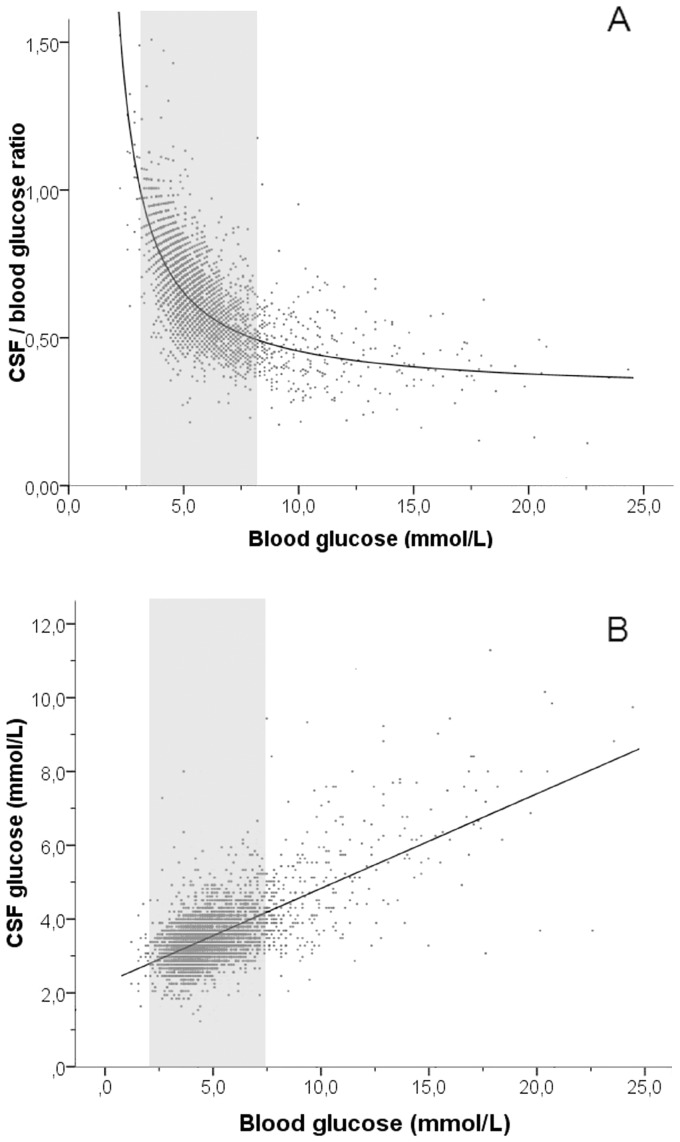
Relation between plasma glucose and CSF glucose. (A) Relation between plasma glucose and CSF/plasma glucose ratio in 4,508 CSF samples. CSF samples with CSF/plasma glucose ratio >1.5 (n = 5) are not shown. (B) Relation between plasma glucose and CSF glucose in 4,513 CSF samples. The grey areas indicates normoglycemia (plasma glucose >3.0 and <7.8 mmol/L).

A linear relation between plasma glucose and CSF glucose was found (Figure 3A). Hypoglycemia was associated with an increased CSF/plasma glucose ratio in most patients, whereas hyperglycemia was associated with only a minimal decrease of the CSF/plasma glucose ratio in the majority of the samples (Figure 3B).

## Discussion

Our study allowed for the first time to define age-specific reference values for CSF glucose, CSF lactate and CSF/plasma glucose for children and adults based on a large number of CSF samples. Reference ranges did not differ between males and females, which is in line with previous findings [15]. To our best knowledge, no studies are available reporting differences in CSF glucose or lactate based on race. We therefore believe that our data, mainly based on CSF samples from Caucasians, can be generalized to non-Caucasians.

Since it seems impossible to obtain CSF from healthy volunteers (especially children) under standardized conditions (e.g. after fasting), we included CSF samples from our computerized database of patients with a medical indication for a lumbar puncture, which reflects the daily clinical practice. Because of the large number of samples it was not feasible to obtain clinical information from the patients. In order to exclude as many as possible CSF samples of patients with overt neurological disorders, we have therefore used strict exclusion criteria based on other CSF parameters (Fig. 1). Furthermore, since the concentrations of CSF glucose and CSF lactate in ventricular CSF differ from lumbar CSF values, we have excluded all ventricular CSF samples.

The reference range for CSF glucose is generally set at 2.5–4.4 mmol/L, but ranges with a lower limit of 2.0 mmol/L or an upper limit of 6.5 mmol/L are used as well [1], [16]. We found an age-dependent pattern for CSF glucose with 5^th^ percentile values ranging from 1.7 to 2.9 mmol/L and 95^th^ percentile values from 4.5 to 5.6 mmol/L. Age-dependency of CSF glucose had been described before in children [17]. CSF glucose 5^th^ percentile values were remarkably low until the age of 6 months (1.7–2.0 mmol/L), which is in line with previous studies [9], [18] (Table 2). The 95^th^ percentile value in our study was especially high in neonates (5.6 mmol/L), which is higher than previously described (3.5–4.0 mmol/L) [18]. Low CSF glucose 5^th^ percentile values and high 95^th^ percentile values in neonates in our study might be related to the high frequency of hypoglycemia and hyperglycemia in neonates, especially in preterm infants and neonates with a low birth weight [19], [20]. After exclusion of CSF samples from patients with abnormal or unknown plasma glucose the 95^th^ percentile values in infants aged 4 to 8 weeks and 3 to 12 months increased more than 10% compared to the original value. This was due to a skewed distribution most likely caused by the low number of CSF samples in these subgroups after exclusion of samples with abnormal or unknown CSF glucose (Table S2).

CSF glucose 5–95^th^ percentile values were relatively high in patients older than 50 years of age. This was probably related to the increasing frequency of hyperglycaemia in patients aged 50 years and older (13.5% compared to 3.7% in patients younger than 50 years of age). In a study comparing the CSF/blood glucose ratio from patients with diabetes mellitus and noninflammatory neurological conditions with patients with bacterial meningitis, the CSF glucose ranged from 2.78 to 25 mmol/L in patients with diabetes, with CSF/blood glucose ratio ranging from 0.20–3.46 [21]. To our best knowledge no studies are available on CSF glucose and lactate values in patients with diabetes without neurological disorders. In order to correct the reference range for CSF glucose and CSF/plasma glucose ratio for patients with abnormal plasma glucose, we have in second instance excluded all patients with abnormal (defined as >7.8 mmol/L or <3.0 mmol/L) or unknown plasma glucose. Reference ranges for CSF glucose before and after correction for plasma glucose are described in Table 1.

The reference value for CSF/plasma glucose ratio is typically about 0.6 and is generally set at 0.5–0.8 [15]. Our study confirms this reference range, although a slight age-dependent pattern for CSF/plasma glucose ratio was found. Below the age of 12 months relatively low 5^th^ percentile values were seen (0.36–0.44), whereas 95^th^ percentiles were high in patients younger than 12 months (1.05–1.20). A high CSF/plasma glucose ratio in newborns is thought to be related to the higher cerebral blood flow in newborns in combination with a smaller brain that utilizes less glucose [22], [23]. Remarkably, the upper reference limit of CSF/plasma glucose values in patients under the age of 12 months in our study exceeds 1.0. Most likely such high ratios are caused by a decline in plasma glucose in the hours before the lumbar puncture, while the concentration in the cerebrospinal fluid follows only gradually. It is known that the equilibration of glucose between plasma and CSF takes approximately two to four hours [24]. Figure 3A shows that a CSF/plasma glucose ratio exceeding 1.0 is predominantly found in patients with hypoglycemia.

A linear correlation was found between plasma glucose (range 2.2–23.3 mmol/L) and CSF glucose (range 1.2–11.0 mmol/L), with CSF glucose values exceeding 8 mmol/L in patients with hyperglycemia. Whereas hypoglycemia was associated with an increase of the CSF/plasma glucose ratio, hyperglycemia barely affected the CSF/plasma glucose ratio (Fig. 3). This finding is in contradiction with the general assumption that CSF glucose concentrations tend to plateau at high blood glucose levels since glucose is transported from blood into CSF by facilitated diffusion and exhibits saturation kinetics [25]. Our study demonstrates that in clinically relevant levels of hyperglycemia (i.e. plasma glucose 7.8–25 mmol/L), CSF glucose is linearly correlated with plasma glucose.

The reference range for CSF lactate is generally set at 1.2–2.1 mmol/L, but ranges from 0.6 to 3.1 mmol/L are used as well (Table 2) [1]. We found a clear age-dependent pattern for CSF lactate. A slight variation with age of the CSF lactate has been described before in a study of children aged 0–15 years [26], with 95^th^ percentile values around 1.95 mmol/L. Higher 95^th^ percentiles for children under the age of 6 months were found in the present study (2.01–2.64 mmol/L). Furthermore, we observed increasing 95^th^ percentile values above the age of 40 years. The tendency for CSF lactate to increase in adulthood (after the age of 54 years) has been described before in a study on cisternal CSF specimens [15].

It is remarkable that after exclusion of all CSF samples with an elevated erythrocyte or leucocyte count, bilirubin, free hemoglobulin, or total protein concentration, 149 CSF samples with a lactate concentrations >3000 µmol/L were found. CSF lactate concentrations can be raised in association with seizures, bactarial, fungal of tuberculous meningitis or encephalitis, cerebral ischemia, neurosarcoidosis, malignancy and metabolic disorders [27]. In most of these disorders, however, other CSF parameters such as leucocyte count and protein count are raised as well. It is possible that some CSF samples from patients with a mitochondrial disorder have been included, which will increase the 95^th^ percentile range of the reference range. We have therefore excluded CSF samples with a CSF lactate concentration >3000 µmol/L in second instance. This, however, did not change the 5–95th percentile value more than10% compared to the original value, except for >10% lower 95^th^ percentile value in patients younger than 4 weeks and patients aged 3–6 months. For these patients we have therefore based the reference range for CSF lactate on the CSF samples with CSF lactate <3000 µmol/L (Table 1).

CSF results for glucose and lactate can be especially helpful in the diagnostic work-up of GLUT1 deficiency syndrome (GLUT1DS), a neurometabolic disorder caused by defective glucose transport into brain. With the exception of rare cases [28]–[30], the biochemical hallmark of GLUT1DS uniformly consists of low CSF glucose and lactate, and low CSF/plasma ratio for glucose [5], [13]. The availability of detailed, age-related reference values as presented here will allow appropriate interpretation of CSF and thereby identification of patients who suffer from this treatable disorder.

In conclusion, we have defined age-specific reference values for CSF glucose, CSF/plasma glucose ratio and CSF lactate. They allow a reliable interpretation of CSF results in everyday clinical practice.

## Supporting Information

Table S1 **N (total) – total number of CSF samples.** N – number of CSF samples with measured CSF glucose concentration. 95% CI – 95% Confidence Interval based on Bootstrap Percentiles (based on 1000 bootstrap samples). Numbers and CSF glucose concentrations between brackets represent the results after exclusion of CSF samples with hypoglycemia (blood glucose <3.0 mmol/L), hyperglycemia (blood glucose >7.8 mmol/L), or unknown blood glucose at the moment of lumbar puncture (only shown if >10% different from the original value).
(DOC)

Table S2 **N (total) – total number of CSF samples.** N – number of CSF samples with measured CSF glucose concentration. 95% CI – 95% Confidence Interval based on Bootstrap Percentiles (based on 1000 bootstrap samples).*) Sample size too small for bootstrapping. Numbers and CSF/blood glucose values between brackets represent the results after exclusion of CSF samples with hypoglycemia (blood glucose <3.0 mmol/L), hyperglycemia (blood glucose >7.8 mmol/L), or unknown blood glucose at the moment of lumbar puncture (only shown if >10% different from the original value).
(DOC)

Table S3 **N (total) – total number of CSF samples.** N – number of CSF samples with measured CSF glucose concentration. 95% CI – 95% Confidence Interval based on Bootstrap Percentiles (based on 1000 bootstrap samples). Numbers and CSF lactate concentrations between brackets represent the results after exclusion of CSF samples with CSF lactate >3000 µmol/L (only shown if >10% different from the original value).
(DOC)
