# Supplementary material for: Cerebrospinal Fluid Glucose and Lactate: Age-Specific Reference Values and Implications for Clinical Practice
Source: PLoS One. 2012 Aug 6;7(8):e42745. doi: 10.1371/journal.pone.0042745 (PMC3412827; doi:10.1371/journal.pone.0042745)
Supplement: Table S1 — N (total) – total number of CSF samples. N – number of CSF samples with measured CSF glucose concentration. 95% CI – 95% Confidence Interval based on Bootstrap Percentiles (based on 1000 bootstrap samples). Numbers and CSF glucose concentrations between brackets represent the results after exclusion of CSF samples with hypoglycemia (blood glucose <3.0 mmol/L), hyperglycemia (blood glucose >7.8 mmol/L), or unknown blood glucose at the moment of lumbar puncture (only shown if >10% different from the original value). (DOC) [file pone.0042745.s001.doc]

| **Table S1. Age-specific CSF glucose concentrations (mmol/L)** | | | | | | | | | | | | | | | | | | | |
| --- | --- | --- | --- | --- | --- | --- | --- | --- | --- | --- | --- | --- | --- | --- | --- | --- | --- | --- | --- |
| **Age** | **N (total)** | **N** | **Mean** | **Median** | **SD** | **Range** | **Percentiles** | |  |  |  | | |  |  |  |  |  |  |
|  |  |  |  |  |  |  | **5** | **95% CI** | | **10** | | **25** | **50** | | **75** | **90** | **95** | **95% CI** |  |
| 0-<4wks | 195 | 190 | 3.40 | 3.1 | 1.16 | 1.3-8.6 | 1.90 | 1.80-2.20 | | 2.20 | | 2.60 | 3.10 | | 4.00 | 5.09 | 5.60 | 5.10-6.60 |  |
| 4-<8wks | 142 | 134 (44) | 2.93 | 2.8 | 1.01 | 1.0-7.8 | 1.68 | 1.40-2.20 | | 2.00 | | 2.30 | 2.80 | | 3.20 | 3.90 | 5.10 (5.65) | 3.96-6.00 |  |
| 8-<12wks | 56 | 56 | 2,89 | 2.9 | 0.648 | 1.4-4.1 | 1.79 | 1.40-2.04 | | 2.00 | | 2.50 | 2.90 | | 3.38 | 3.80 | 4.00 | 3.73-4.10 |  |
| 3-<6mo | 103 | 101 (28) | 2.97 | 3.0 | 0.682 | 1.6-5.8 | 2.00 | 2.00-2.24 | | 2.22 | | 2.60 | 3.00 | | 3.20 | 3.68 | 3.90 (4.90) | 3.60-5.60 |  |
| 6-<12mo | 196 | 192 (43) | *3,23* | 3.2 | 0.596 | 1.6-5.6 | 2.40 | 2.20-2.60 | | 2.60 | | 2.80 | 3.20 | | 3.50 | 4.00 | 4.30 (4.88) | 4.00-4.80 |  |
| 1-<2yrs | 368 | 362 | 3.20 | 3.1 | 0.626 | 1.8-9.1 | 2.40 | 2.40-2.50 | | 2.60 | | 2.80 | 3.10 | | 3.43 | 3.90 | 4.20 | 4.10-4.40 |  |
| 2-<3yrs | 306 | 302 (95) | 3.09 | 3.0 | 0.542 | 2.0-5.7 | 2.40 | 2.30-2.45 | | 2.60 | | 2.80 | 3.00 | | 3.30 | 3.80 (3.40) | 4.20 | 4.00-4.40 |  |
| 3-<4yrs | 328 | 321 | 3.09 | 3.0 | 0.527 | 1.4-5.9 | 2.40 | 2.30-2.50 | | 2.60 | | 2.80 | 3.00 | | 3.30 | 3.70 | 3.80 | 3.80-4.20 |  |
| 4-<5yrs | 306 | 298 (99) | 3.10 | 3.0 | 0.512 | 2.2-5.6 | 2.40 | 2.30-2.50 | | 2.60 | | 2.80 | 3.00 | | 3.30 | 3.70 (4.20) | 4.11 | 3.83-4.40 |  |
| 5-<10yrs | 929 | 913 | 3.17 | 3.1 | 0.509 | 1.8-7.3 | 2.50 | 2.50-2.60 | | 2.60 | | 2.80 | 3.10 | | 3.40 | 3.80 | 4.00 | 3.90-4.15 |  |
| 10-<18yrs | 800 | 781 | 3.31 | 3.2 | 0.630 | 1.9-8.8 | 2.60 | 2.50-2.60 | | 2.70 | | 3.00 | 3.20 | | 3.50 | 3.90 | 4.30 | 4.10-4.50 |  |
| 18-<30yrs | 610 | 601 | 3.40 | 3.3 | 0.662 | 1.8-8.6 | 2.70 | 2.60-2.80 | | 2.80 | | 3.10 | 3.30 | | 3.60 | 4.00 | 4.40 | 4.20-4.70 |  |
| 30-<40yrs | 774 | 754 | 3.41 | 3.3 | 0.677 | 1.0-10.1 | 2.70 | 2.60-2.80 | | 2.90 | | 3.10 | 3.30 | | 3.60 | 3.90 | 4.30 | 4.00-4.60 |  |
| 40-<50yrs | 1069 | 1042 | 3.49 | 3.4 | 0.729 | 1.4-10.5 | 2.80 | 2.70-2.80 | | 2.90 | | 3.10 | 3.40 | | 3.70 | 4.04 | 4.40 | 4.20-4.70 |  |
| 50-<60yrs | 1120 | 1108 (624) | 3.58 | 3.5 | 0.668 | 2.0-8.9 | 2.80 | 2.70-2.80 | | 3.00 | | 3.20 | 3.50 | | 3.80 | 4.20 | 4.80 (4.20) | 4.60-5.20 |  |
| 60-<70yrs | 924 | 914 (420) | 3.74 | 3.5 | 0.901 | 1.8-8.9 | 2.80 | 2.80-2.90 | | 3.00 | | 3.20 | 3.50 | | 3.90 | 4.70 (4.09) | 5.60 (4.40) | 5.20-6.00 |  |
| 70-<80yrs | 631 | 624 (253) | 3.85 | 3.6 | 0.984 | 1.6-11.0 | 2.90 | 2.80-3.00 | | 3.00 | | 3.30 | 3.60 | | 4.10 | 5.00 (4.30) | 5.90 (4.50) | 5.30-6.40 |  |
| ≥80yrs | 177 | 176 (56) | 3.88 | 3.6 | 1.154 | 2.3-11.9 | 2.89 | 2.60-3.00 | | 3.00 | | *3.20* | 3.60 | | 4.20 | 4.93 (4.40) | 6.12 (4.50) | 5.03-6.94 |  |
| **Total** | **9036** | **8871 (4173)** | **3.42** | **3.3** | **0.769** | **1.0-11.9** | **2.60** | **2.50-2.60** | | **2.70** | | **3.00** | **3.30** | | **3.70** | **4.20** | **4.70 (4.20)** | **4.60-4.80** |  |
|  | | | | | | | | | | | | | | | | | | |  |
