# Supplementary material for: Cerebrospinal Fluid Glucose and Lactate: Age-Specific Reference Values and Implications for Clinical Practice
Source: PLoS One. 2012 Aug 6;7(8):e42745. doi: 10.1371/journal.pone.0042745 (PMC3412827; doi:10.1371/journal.pone.0042745)
Supplement: Table S2 — N (total) – total number of CSF samples. N – number of CSF samples with measured CSF glucose concentration. 95% CI – 95% Confidence Interval based on Bootstrap Percentiles (based on 1000 bootstrap samples).*) Sample size too small for bootstrapping. Numbers and CSF/blood glucose values between brackets represent the results after exclusion of CSF samples with hypoglycemia (blood glucose <3.0 mmol/L), hyperglycemia (blood glucose >7.8 mmol/L), or unknown blood glucose at the moment of lumbar puncture (only shown if >10% different from the original value). (DOC) [file pone.0042745.s002.doc]

| **Table S2. Age-specific CSF/blood glucose ratio** | | | | | | | | | | | | | | | | | | | | | | | |
| --- | --- | --- | --- | --- | --- | --- | --- | --- | --- | --- | --- | --- | --- | --- | --- | --- | --- | --- | --- | --- | --- | --- | --- |
| **Age** | **N (total)** | **N** | **Mean** | **Median** | **SD** | **Range** | **Percentiles** |  |  | |  | |  | | |  | |  | |  | |  |  |
|  |  |  |  |  |  |  | **5** | **95% CI** | | **10** | | **25** | | **50** | **75** | | **90** | | **95** | | **95% CI** | |  |
| 0-<4wks | 195 | 48 (44) | 0.72 | 0.67 | 0.27 | 0.37-1.62 | 0.42 | 0.37-0.46 | | 0.45 | | 0.52 | | 0.67 | 0.83 | | 1.10 | | 1.38 (1.10) | | 1.03-1.63 | |  |
| 4-<8wks | 142 | 47 (44) | 0.65 | 0.58 | 0.27 | 0.24-1.77 | 0.36 | 0.24-0.46 | | 0.43 | | 0.49 | | 0.58 | 0.77 | | 0.98 (0.87) | | 1.19 | | 0.87-1.77 | |  |
| 8-<12wks | 56 | 11 (10) | 0.64 | 0.64 | 0.18 | 0.40-1.03 | 0.40 | 0.40-0.57 | | 0.41 | | 0.49 | | 0.64 | 0.76 | | 0.99 | | 1.03 | | * | |  |
| 3-<6mo | 103 | 29 | 0.60 | 0.57 | 0.20 | 0.33-1.45 | 0.37 | 0.33-0.47 | | 0.46 | | 0.49 | | 0.57 | 0.65 | | 0.76 | | 1.16 | | 0.68-1.45 | |  |
| 6-<12mo | 196 | 46 | 0.64 | 0.59 | 0.16 | 0.43-1.23 | 0.44 | 0.43-0.49 | | 0.47 | | 0.54 | | 0.59 | 0.70 | | 0.82 | | 1.05 | | 0.78-1.23 | |  |
| 1-<2yrs | 368 | 173 | 0.64 | 0.65 | 0.13 | 0.17-1.00 | 0.44 | 0.39-0.47 | | 0.49 | | 0.56 | | 0.65 | 0.73 | | 0.80 | | 0.87 | | 0.82-0.90 | |  |
| 2-<3yrs | 306 | 102 | 0.63 | 0.64 | 0.12 | 0.18-1.03 | 0.43 | 0.26-0.51 | | 0.51 | | 0.58 | | 0.64 | 0.69 | | 0.78 | | 0.82 | | 0.78-0.91 | |  |
| 3-<4yrs | 328 | 106 | 0.64 | 0.64 | 0.13 | 0.19-0.90 | 0.43 | 0.38-0.46 | | 0.46 | | 0.55 | | 0.64 | 0.74 | | 0.81 | | 0.86 | | 0.81-0.88 | |  |
| 4-<5yrs | 306 | 105 | 0.63 | 0.62 | 0.15 | 0.22-1.47 | 0.45 | 0.22-0.49 | | 0.49 | | 0.56 | | 0.62 | 0.72 | | 0.78 | | 0.83 | | 0.78-0.91 | |  |
| 5-<10yrs | 929 | 342 | 0.64 | 0.63 | 0.12 | 0.27-1.12 | 0.47 | 0.45-0.48 | | 0.50 | | 0.56 | | 0.63 | 0.71 | | 0.79 | | 0.85 | | 0.81-0.91 | |  |
| 10-<18yrs | 800 | 276 | 0.65 | 0.64 | 0.13 | 0.26-1.29 | 0.47 | 0.41-0.49 | | 0.50 | | 0.57 | | 0.64 | 0.72 | | 0.81 | | 0.83 | | 0.81-0.91 | |  |
| 18-<30yrs | 610 | 401 | 0.68 | 0.67 | 0.14 | 0.25-1.50 | 0.46 | 0.44-0.49 | | 0.52 | | 0.60 | | 0.67 | 0.75 | | 0.83 | | 0.90 | | 0.85-0.97 | |  |
| 30-<40yrs | 774 | 536 | 0.68 | 0.67 | 0.13 | 0.30-1.59 | 0.47 | 0.45-0.50 | | 0.52 | | 0.61 | | 0.67 | 0.74 | | 0.83 | | 0.90 | | 0.86-0.92 | |  |
| 40-<50yrs | 1069 | 726 | 0.65 | 0.65 | 0.13 | 0.23-1.21 | 0.46 | 0.44-0.48 | | 0.50 | | 0.58 | | 0.65 | 0.71 | | 0.80 | | 0.88 | | 0.84-0.91 | |  |
| 50-<60yrs | 1120 | 685 (624) | 0.65 | 0.65 | 0.14 | 0.24-1.60 | 0.43 (0.48) | 0.42-0.46 | | 0.48 (0.53) | | 0.57 | | 0.65 | 0.73 | | 0.81 | | 0.87 | | 0.84-0.90 | |  |
| 60-<70yrs | 924 | 496 (420) | 0.63 | 0.62 | 0.13 | 0.31-1.04 | 0.42 (0.46) | 0.40-0.44 | | 0.46 (0.51) | | 0.55 | | 0.62 | 0.71 | | 0.80 | | 0.85 | | 0.82-0.87 | |  |
| 70-<80yrs | 631 | 312 (253) | 0.62 | 0.61 | 0.16 | 0.30-2.03 | 0.39 (0.47) | 0.36-0.41 | | 0.43 (0.51) | | 0.52 | | 0.61 | 0.71 | | 0.79 | | 0.84 | | 0.80-0.88 | |  |
| ≥80yrs | 179 | 75 (56) | 0.56 | 0.57 | 0.13 | 0.24-0.87 | 0.35 (0.42) | 0.26-0.41 | | 0.40 (0.49) | | 0.48 | | 0.57 | 0.63 | | 0.74 | | 0.81 | | 0.72-0.86 | |  |
| **Total** | **9036** | **4516** | **0.65** | **0.64** | **0.14** | **0.17-2.03** | **0.44** | **0.43-0.45** | | **0.49** | | **0.57** | | **0.64** | **0.72** | | **0.81** | | **0.87** | | **0.86-0.88** | |  |
| N (total) – total number of CSF samples. N – number of CSF samples with measured CSF glucose concentration. 95% CI – 95% Confidence Interval based on Bootstrap Percentiles (based on 1000 bootstrap samples). *) Sample size too small for bootstrapping. Numbers and CSF/blood glucose values between brackets represent the results after exclusion of CSF samples with hypoglycemia (blood glucose <3.0 mmol/L), hyperglycemia (blood glucose >7.8 mmol/L), or unknown blood glucose at the moment of lumbar puncture (only shown if >10% different from the original value). | | | | | | | | | | | | | | | | | | | | | | |  |
