# Supplementary material for: Cerebrospinal Fluid Glucose and Lactate: Age-Specific Reference Values and Implications for Clinical Practice
Source: PLoS One. 2012 Aug 6;7(8):e42745. doi: 10.1371/journal.pone.0042745 (PMC3412827; doi:10.1371/journal.pone.0042745)
Supplement: Table S3 — N (total) – total number of CSF samples. N – number of CSF samples with measured CSF glucose concentration. 95% CI – 95% Confidence Interval based on Bootstrap Percentiles (based on 1000 bootstrap samples). Numbers and CSF lactate concentrations between brackets represent the results after exclusion of CSF samples with CSF lactate >3000 µmol/L (only shown if >10% different from the original value). (DOC) [file pone.0042745.s003.doc]

| **Table S3. Age-specific CSF lactate concentrations (µmol/L)** | | | | | | | | | | | | | | | | | | | | |
| --- | --- | --- | --- | --- | --- | --- | --- | --- | --- | --- | --- | --- | --- | --- | --- | --- | --- | --- | --- | --- |
| **Age** | **N (total)** | **N** | **Mean** | **Median** | **SD** | **Range** | **Percentiles** |  |  | |  | |  | |  |  | |  |  |  |
|  |  |  |  |  |  |  | **5** | **95% CI** | | **10** | **25** | **50** | | **75** | **90** | | **95** | | **95% CI** |  |
| 0-<4wks | 195 | 156 (145) | 1750 | 1510 | 911 | 763- 6550 | 882 | 832-988 | | 995 | 1239 | 1510 | | 1898 | 2635 (2203) | | 3379 (2484) | | 2690-5080 |  |
| 4-<8wks | 142 | 107 | 1511 | 1472 | 434 | 695-3094 | 916 | 755-1028 | | 1028 | 1239 | 1472 | | 1708 | 2024 | | 2164 | | 2029-3021 |  |
| 8-<12wks | 56 | 50 | 1473 | 1366 | 493 | 817-3825 | 861 | 817-1104 | | 1068 | 1159 | 1366 | | 1633 | 2006 | | 2352 | | 1887-3825 |  |
| 3-<6mo | 103 | 89 (84) | 1608 | 1426 | 770 | 822-6700 | 1001 | 933-1147 | | 1143 | 1222 | 1426 | | 1627 | 2136 | | 3341 (2135) | | 2132-4195 |  |
| 6-<12mo | 196 | 151 | 1509 | 1453 | 430 | 874-4623 | 1124 | 1059-1156 | | 1168 | 1304 | 1453 | | 1598 | 1814 | | 2170 | | 1842-2530 |  |
| 1-<2yrs | 368 | 301 | 1470 | 1380 | 523 | 732-5385 | 990 | 950-1069 | | 1086 | 1241 | 1380 | | 1598 | 1792 | | 1997 | | 1851-2397 |  |
| 2-<3yrs | 306 | 231 | 1438 | 1362 | 447 | 642-5434 | 989 | 910-1028 | | 1093 | 1207 | 1362 | | 1570 | 1800 | | 2015 | | 1864-2223 |  |
| 3-<4yrs | 328 | 225 | 1422 | 1328 | 452 | 705-5970 | 1037 | 1000-1077 | | 1098 | 1199 | 1328 | | 1535 | 1802 | | 2031 | | 1848-2325 |  |
| 4-<5yrs | 306 | 174 | 1474 | 1383 | 482 | 440-4320 | 998 | 924-1090 | | 1110 | 1224 | 1383 | | 1603 | 1919 | | 2259 | | 2011-2781 |  |
| 5-<10yrs | 929 | 510 | 1528 | 1453 | 428 | 811-5120 | 1140 | 1120-1171 | | 1195 | 1306 | 1453 | | 1622 | 1833 | | 2097 | | 1960-2442 |  |
| 10-<18yrs | 800 | 517 | 1605 | 1536 | 398 | 925-5300 | 1198 | 1165-1222 | | 1258 | 1378 | 1536 | | 1709 | 1991 | | 2243 | | 2106-2500 |  |
| 18-<30yrs | 610 | 577 | 1619 | 1553 | 435 | 992-7600 | 1223 | 1189-1250 | | 1289 | 1409 | 1553 | | 1718 | 1946 | | 2200 | | 2102-2337 |  |
| 30-<40yrs | 774 | 755 | 1661 | 1595 | 346 | 156-3813 | 1280 | 1230-1311 | | 1347 | 1466 | 1595 | | 1772 | 2001 | | 2310 | | 2209-2434 |  |
| 40-<50yrs | 1069 | 1026 | 1749 | 1667 | 494 | 350-12598 | 1358 | 1338-1378 | | 1413 | 1517 | 1667 | | 1870 | 2131 | | 2410 | | 2270-2497 |  |
| 50-<60yrs | 1120 | 1084 | 1803 | 1714 | 453 | 1111-6580 | 1332 | 1309-1363 | | 1423 | 1546 | 1714 | | 1931 | 2216 | | 2500 | | 2404-2666 |  |
| 60-<70yrs | 924 | 879 | 1854 | 1760 | 452 | 572-6056 | 1386 | 1348-1419 | | 1456 | 1598 | 1760 | | 1985 | 2280 | | 2624 | | 2458-2900 |  |
| 70-<80yrs | 631 | 604 | 1887 | 1809 | 389 | 261-4000 | 1408 | 1386-1444 | | 1504 | 1627 | 1809 | | 2070 | 2330 | | 2626 | | 2449-2722 |  |
| ≥80yrs | 177 | 176 | 1924 | 1853 | 386 | 1129-3556 | 1411 | 1294-1484 | | 1492 | 1667 | 1853 | | 2104 | 2437 | | 2708 | | 2500-2851 |  |
| **Total** | **9036** | **7614** | **1693** | **1615** | **481** | **156-12598** | **1176** | **1164-1190** | | **1276** | **1432** | **1615** | | **1841** | **2138** | | **2423** | | **2384-2455** |  |
|  | | | | | | | | | | | | | | | | | | | |  |
